# A novel CO_2_ utilization technology for the synergistic co-production of multi-walled carbon nanotubes and syngas

**DOI:** 10.1038/s41598-021-80986-2

**Published:** 2021-01-14

**Authors:** Mohamed S. Challiwala, Hanif A. Choudhury, Dingdi Wang, Mahmoud M. El-Halwagi, Eric Weitz, Nimir O. Elbashir

**Affiliations:** 1 Artie Mcferrin Department of Chemical Engineering, Texas A&M University, College Station, USA; 2 TEES Gas and Fuels Research Center, College Station, USA; 3 Chemical Engineering and Petroleum Engineering Program, Texas A&M University at Qatar, Doha, Qatar; 4 Northwestern University, Evanston, IL USA

**Keywords:** Chemical engineering, Heterogeneous catalysis

## Abstract

Dry reforming of methane (DRM) is a well-known process in which CH_4_ and CO_2_ catalytically react to produce syngas. Solid carbon is a well-known byproduct of the DRM but is undesirable as it leads to catalyst deactivation. However, converting CO_2_ and CH_4_ into solid carbon serves as a promising carbon capture and sequestration technique that has been demonstrated in this study by two patented processes. In the first process, known as CARGEN technology (CARbon GENerator), a novel concept of two reactors in series is developed that separately convert the greenhouse gases (GHGs) into multi-walled carbon nanotubes (MWCNTs) and syngas. CARGEN enables at least a 50% reduction in energy requirement with at least 65% CO_2_ conversion compared to the DRM process. The second process presents an alternative pathway for the regeneration/reactivation of the spent DRM/CARGEN catalyst using CO_2_. Provided herein is the first report on an experimental demonstration of a 'switching' technology in which CO_2_ is utilized in both the operation and the regeneration cycles and thus, finally contributing to the overall goal of CO_2_ fixation. The following studies support all the results in this work: physisorption, chemisorption, XRD, XPS, SEM, TEM, TGA, ICP, and Raman analysis.

## Introduction

Syngas production via DRM is being considered as an attractive CO_2_ conversion process. However, its industrial implementation is still subject to the development of more mature and advanced technology. The limitations of the DRM process can be attributed to the following: high endothermicity of the reaction; low syngas quality (i.e., H_2_: CO ≤ 1); and extremely high coking tendency that creates technological hurdles with prohibitive economic solutions. In contrast to the commercial technologies like: steam methane reforming (SRM), partial oxidation (POx), and auto-thermal reforming (ATR), the main benefit of DRM, however, is its ability to utilize CO_2_. The downstream plants like Fischer Tropsch (FTS) or methanol synthesis require an H_2_/CO ratio of 2:1, which cannot be fulfilled by the quality of the syngas produced from the DRM unit. To address this challenge, a study by Olah et al.^1^ reported a combination of SRM and DRM over NiO/MgO catalysts to produce an H_2_/CO ratio of 2:1, which is compatible with most of the downstream gas to liquid (GTL) facilities. However, a combination of DRM and SRM is less sustainable as both the processes are highly endothermic, resulting in significant indirect CO_2_ emissions. In this regard, life cycle assessment (LCA) presents a very promising technique in identifying and accounting for all the direct and indirect emissions in a particular process^2^. Regarding the second challenge of carbon formation, the accomplishments from the field of catalysis have resulted in the development of superior catalytic systems showcasing remarkable resistance towards carbon formation^3–11^. However, the third challenge of high endothermicity still remains, in addition to the fact that all the three challenges have never been addressed in a single process. Therefore, the research on the DRM process has always been active in the search for a radically new process targeting all the three challenges of DRM that would enable its commercial implementation.

**Figure 1:**
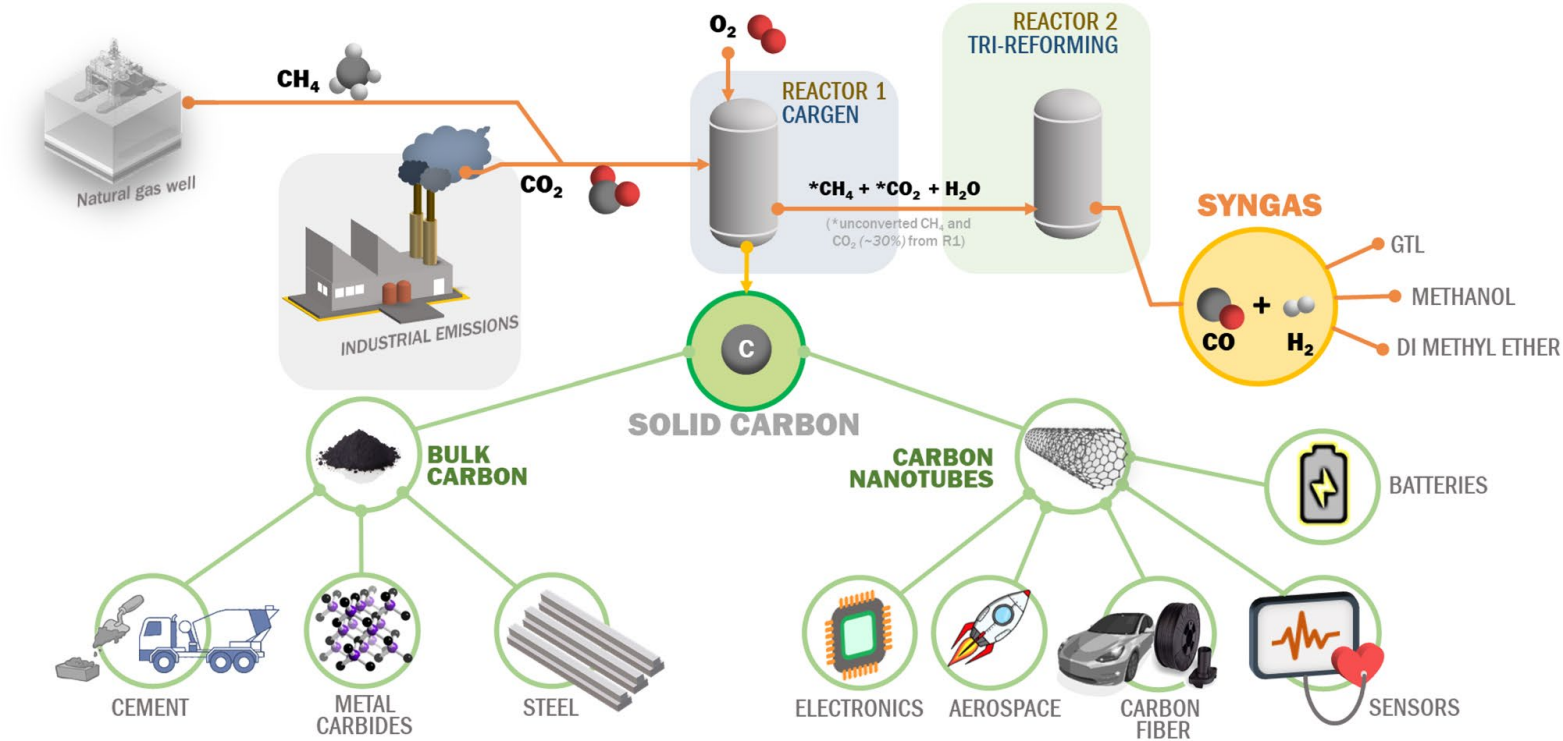
A systematic overview of the novel two-reactor technology with potential applications of the products formed.

In this communication, reported is an experimental demonstration of a combination of two novel processes that enable DRM implementation^12,13^. *The first process* is a combination of two reactors in series, wherein the first reactor (also known as CARGEN or CARbon GENerator) converts CO_2_ and CH_4_ to multi-walled carbon nanotubes (MWCNTs). While the other reactor produces syngas with a desirable H_2_/CO ratio that meets downstream requirements. A systematic overview of the CARGEN process is provided in Fig. 1, and more details on its concept are provided in supplementary Section 1. *The second process*, on the other hand, utilizes CO_2_ during the catalyst regeneration cycle for the removal of surface carbon. A combination of CARGEN and the new CO_2_ regeneration process forms a switching technology that utilizes CO_2_ during both operations as well as the regeneration cycle.

## Results and discussion

**Figure 2:**
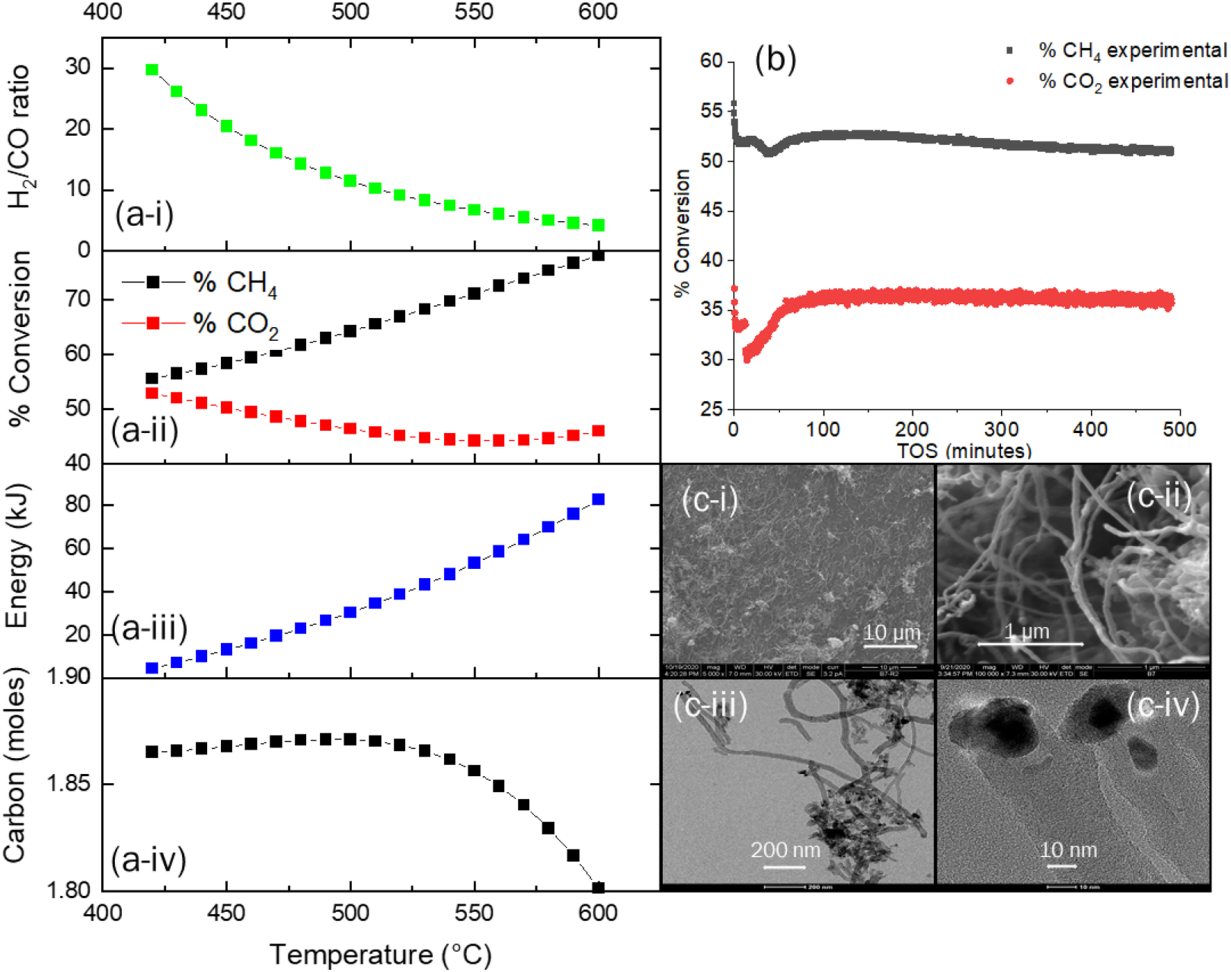
(**a**) Thermodynamic profiles of the novel CARGEN reactor from 400 to 600 °C temperature, (i) Syngas ratio at CH_4_:CO_2_:O_2_ feed ratio of 1:/0.6/0.1. (ii) % Conversion of reactant gases. (iii) Energy requirement at reported temperature conditions, (iv) carbon formation tendency in moles at reported temperature conditions. (**b**) % Experimental conversions vs. time on stream (TOS) in a flow through reactor for a TOS of 500 min at 550 °C using 20 wt% Ni/Al_2_O_3_ catalyst (**c**-i, **c**-ii): SEM images at 10 μm and 1 μm respectively, (**c**-iii, **c**-iv): TEM images at 200 nm and 10 nm, respectively. Please note that the dotted lines in Fig. 2 a represent the temperature condition at which experiments were conducted that are reported in (**b**).

The thermodynamic equilibrium assessment of the CARGEN reactor shows that solid carbon is a favorable product in a temperature range of 420 °C to 600 °C as shown in Fig. 2a. The feed gas composition was CH_4_/CO_2_/O_2_ = 1/0.6/0.1, that closely resembles flue/landfill/biogas composition^14^ for ultimate FTS application^15^. However, this feed ratio may be changed depending upon available feed gas at site to get different results, and an optimization can also be done to achieve a desired quantity of carbon, or achieve a specific syngas ratio. Figure 2b shows the experimental conversion profile of the CARGEN reactor at a temperature of 550 °C, which closely matches with the theoretical results reported in Fig. 2a-ii. It should be noted that all the experimental studies in this work were conducted using commercially available 20% Ni/Al_2_O_3_ catalyst, as it is the most widely used reforming catalyst. However, other materials like Fe, Co, Mo, Ru, Pt, Rh etc. supported with SiO_2_, TiO_2_ etc. could be used, but will result in higher cost and sintering challenges. It can be seen in Fig. 2 that experimental results closely match thermodynamic results within a margin of 10–15%. It is important to note that the experimental conversions are lower compared to the estimated thermodynamic conversion due to kinetics and mass transfer limitations that arise during the catalytic reaction^16^. A closer examination of the actual spent catalyst from this experimental run using SEM and TEM (Fig. 2c) reveals the formation of MWCNTs.

**Figure 3:**
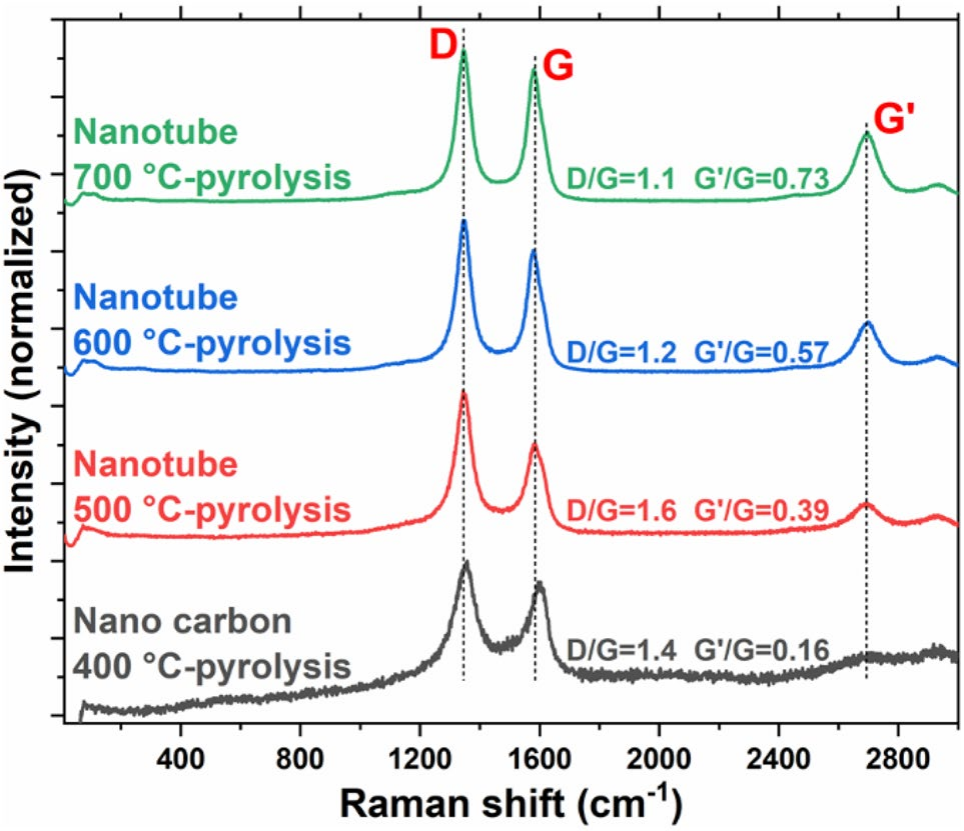
Raman spectra of surface carbon formed from 30-min CH_4_ pyrolysis at 400, 500, 600 and 700 °C respectively on 200 mg 20 wt% Ni/Al_2_O_3_ at a flow rate of 30 ml/min. The ratio of the intensity of D/G peaks is a measure of the defects present on carbon nanomaterials.

Raman analysis of a 30-min CH_4_ pyrolysis experiment on 20 wt% Ni/Al_2_O_3_ at 700, 600, 500, and 400 °C showed that the MWCNT formation regime is gradually diminished with a decrease in temperature, and only nano-carbon remains at 400 °C. These results demonstrate that MWCNT begins forming after between 400 and 500 °C. Figure 3 illustrates the corresponding ex-situ micro-Raman spectra, which indicates that the quality of the MWCNT gets better (D/G ratio decreases while G'/G ratio increases) with an increase in temperature. It should be noted that the operational window of CARGEN is in the temperature range of 400–600 °C, which is the most suitable condition for MWCNT formation per Raman results. High-value products coming from GHGs have attracted significant media attention^17–20^ as well as interest from global energy corporations due to its encouraging preliminary economic data^21^. The commercial value of high-quality MWCNTs can be in the range of 500–10,000 USD/kilogram^22^. Although a detailed techno-economic assessment is underway, the preliminary life cycle assessment (LCA) study revealed that both the CO_2_ footprint and the operational cost of the CARGEN process are approx. 40% that of conventional ATR processes^21^.

**Figure 4:**
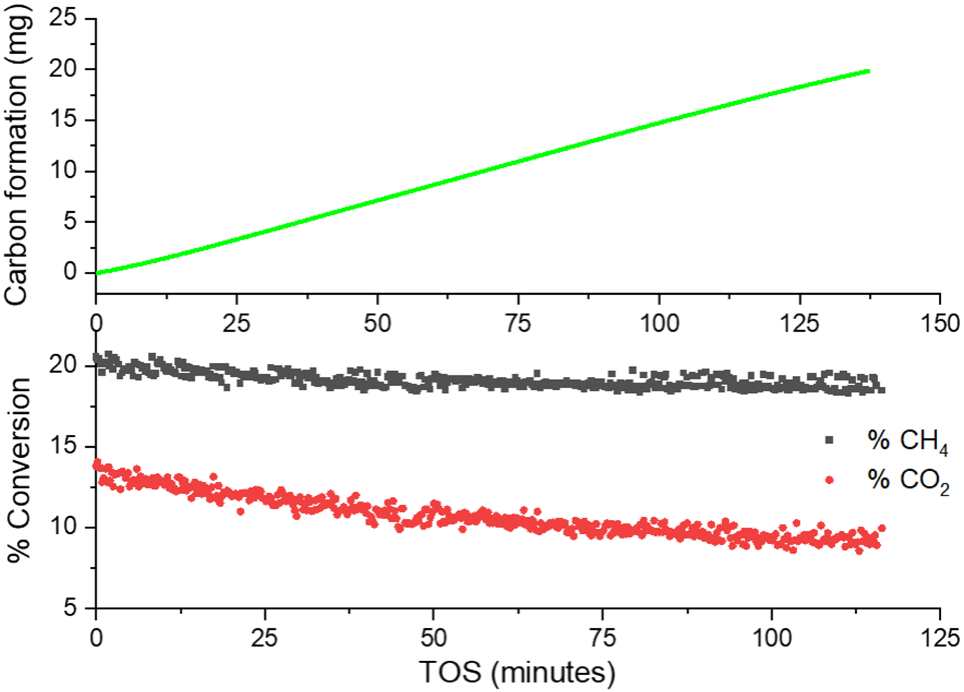
Carbon growth vs. TOS on 20 mg of commercial 20% Ni/γ-Al_2_O_3_ catalyst and its corresponding conversion profiles of CH_4_ and CO_2_.

A thermo-gravimetric analysis (TGA) experiment coupled with material balance on residual gas analyzer (RGA) data enabled direct assessment of the carbon formation rate and feed conversions as a function of TOS. These results are provided in Fig. 4 and detailed calculation steps of material balance in supplementary Sect. 3. Around 20 mg carbon formation was observed in 138 min TOS at 550 °C on 20 mg of commercial 20 wt% Ni/γ-Al_2_O_3_ catalyst entailing a remarkable carbon formation rate of 0.00722 mg_MWCNT_/mg_Cat._/min. It should be reiterated that the target for CARGEN reactor (the first reactor in CARGEN technology) is only to produce solid carbon. The second reactor on the other hand, which is a combined reformer offers a great flexibility in producing a desirable syngas ratio as discussed in detail in previous publications^12,23^.

**Figure 5:**
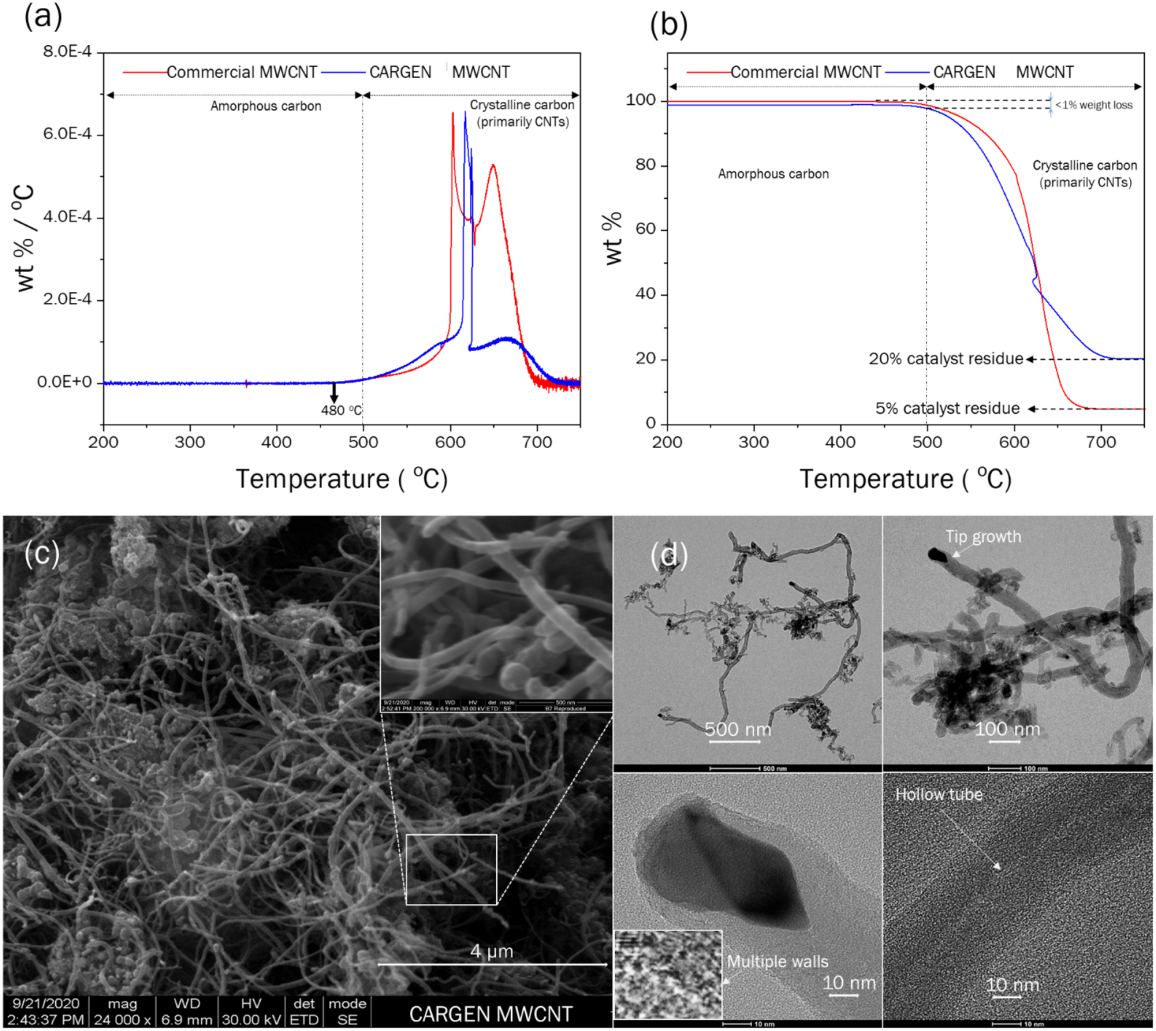
(**a**) Differential Thermogravimetric profiles of the MWCNTs produced from CARGEN and from commercial source, (**b**) Weight loss profiles of the commercial MWCNTs and the CARGEN produced MWCNTs, (**c**) SEM micrograph showing the length and diameter of the MWCNTs produced from CARGEN, (**d**) TEM images of the MWCNTs showing tip-growth mechanism and the multi-walled hollow characteristic of the as-produced MWCNTs from CARGEN.

Subsequent to the production of milli-grams of carbon in TGA, a larger batch of solid carbon was produced in a chemical vapor deposition (CVD) setup to produce multi-grams of carbon. One of the sample produced from this experiment was at ~ 80 wt% carbon purity (and remaining catalyst weight). In order to deduce the quality of the as-produced samples, a TGA air oxidation experiment was then conducted. The protocol for this test involved air combustion of carbon at 50 mL/min in the TGA on ca. 10 mg of the sample under a temperature ramp of 10 °C/min from room temperature to 400 °C, and at 5 °C/min from 400 °C to 800 °C. Figure 5a,b shows that only < 1% of the sample was oxidized between 200 and 500 °C, which is the literature reported range of amorphous carbon^24–26^. As the combustion occured in the range of 480 to 700 °C temperature indicates that crystalline form of carbon was primarily present, of which MWCNTs are predominant category^24–26^. Consolidating the TGA information to the SEM and TEM micrographs infers that most of the carbon belongs to MWCNT category with diameters in the range of 50 to 100 nm and length in the range of 10 to 30 µm as shown in Fig. 5. Moreover, the HR-TEM images at 10 nm scale bar shows that a tip-growth mechanism of CNT production would have taken place during the CARGEN process.

One of the primary challenges in any industrial process that involves carbon formation like DRM, or catalytic cracker units is catalyst deactivation. Due to this, the entire plant has to be taken into maintenance, and the bulk of carbon has to be removed by either scraping or by using other carbon removal techniques like etching, sonication, etc. However, even after the removal of bulk carbon, the catalyst nano sites are still covered with surface carbon^27^, which needs to be removed to reactivate the catalyst. In this paper, a novel approach of a single-step catalyst regeneration procedure, applicable for any process that suffers from catalyst deactivation via coke formation^17^ is presented. The said process utilizes CO_2_ as a soft oxidant^28^ for the removal of surface carbon that deactivated the catalyst. This presents a great improvement over conventional procedure, as the conventional procedure requires two steps- first, oxidation with O_2_ for carbon removal as CO_2_, followed by the reduction of nickel oxide with H_2_ to produce active nickel. The new procedure is precisely similar to temperature-programmed oxidation (TPO) using O_2_ but utilizes CO_2_ in its place. The reaction is proposed to happen via reverse-boudouard route as follows:

1 $${\text{CO}}_{{2}} + {\text{C}} \to {\text{2CO}}$$

**Figure 6:**
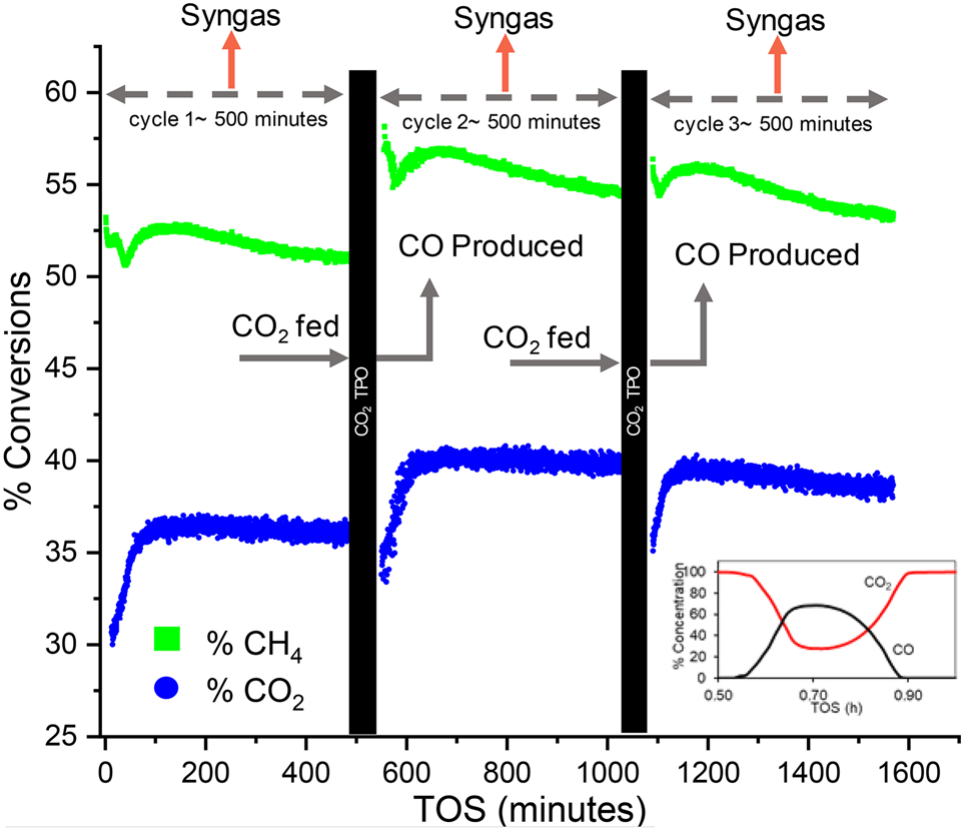
Three cycles of repeatability of the new regeneration technique demonstrating that there is no need for reduction after every surface carbon removal step using CO_2_. Inset figure: Concentration plot from RGA showing the release of CO during CO_2_ TPO. Conditions: 200 mg 20% Ni/γ-Al_2_O_3_, flow: 94 mL/min, T: 550 °C.

This reaction was first tested using thermodynamic equilibrium assessment at temperatures in the range of 650 to 800 °C, followed by laboratory proof of concept study in a flow-through reactor. Under a temperature ramp from 650 to 800 °C, when the CO_2_ gas was passed through the spent DRM catalyst bed, it reacted with surface carbon and produced CO (which could be used as a precursor for several industrial chemicals). A subsequent DRM immediately after the regeneration showed activity without the need for a second reduction step by H_2_. Figure 6 shows the results of this test for three cycles that ensures repeatability. A combination of the operation and regeneration cycle as in Fig. 6 demonstrates an interesting switching process wherein uninterrupted CO_2_ utilization happens during both the cycles.

**Figure 7:**
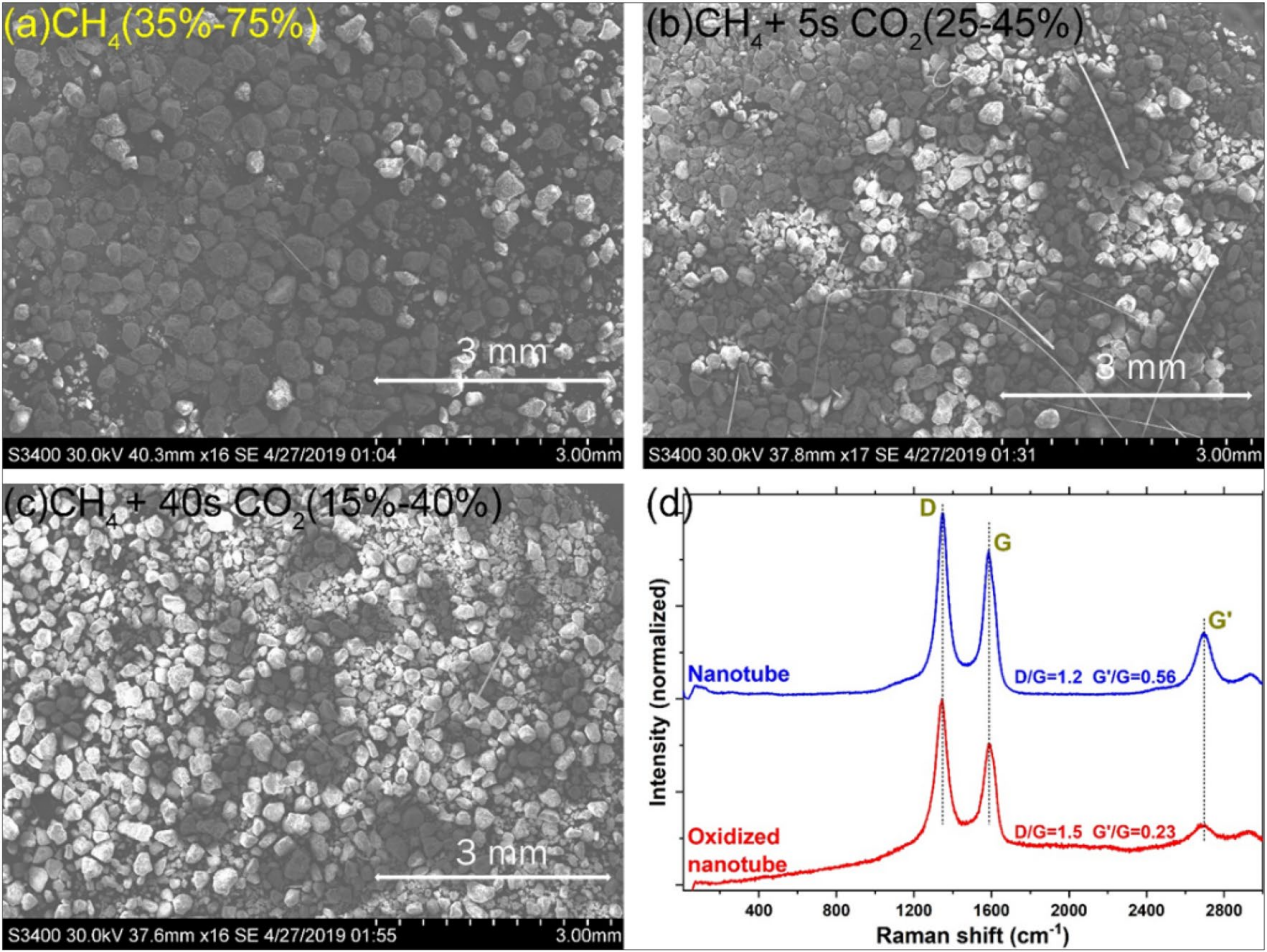
SEM images of (**a**) surface carbon deposited continuously from room temperature to 700 °C during CH_4_ pyrolysis, which undergoes oxidation by (**b**) a 5 s-CO_2_ pulse or (**c**) a 40 s CO_2_ pulse. (**d**) Raman spectra of MWCNT without and with the 40 s-CO_2_ treatment.

In order to evaluate the CO_2_ oxidation capability, pre-reduced 20 wt% Ni/Al_2_O_3_ catalyst was heated continuously from room temperature to 700 °C in CH_4_, then CH_4_ supply was cut off and one CO_2_ pulse was injected for 5 s and for 40 s respectively in two independent experiments. Figure 7 shows the SEM and Raman results of this study. It was observed that the quality of the CO_2_-treated MWCNTs becomes worse (D/G ratio increases while G'/G ratio decreases). It seems that the more defective carbon structures are preferentially removed from the Ni surface while the robust ones remain on the surface with more defects.

Results of XPS analysis of a coked commercial catalyst showed that the surface carbon atom percentage was about 75%. On the other hand, the surface carbon coverage of post-CO_2_ TPO treated catalyst was about 5%, while for post O_2_ TPO was 4.5%. This shows the equivalency of both the regeneration techniques in removing surface carbon. It should be emphasized that even though pure CO_2_ is required for CO_2_ TPO, it has a greater importance for DRM since its use will enable overall CO_2_ fixation.

In terms of sustainability benefits, it was observed that, the new CO_2_ TPO process is capable of converting at least 3 kg of CO_2_ per kg of surface carbon removed, while O_2_ TPO leads to about 0.5 kg of CO_2_ emission per kg of surface carbon. Detailed calculation steps are provided in Sect. 5 of the Supplementary Information. It should be emphasized that the use of CO_2_ regeneration technique also saves the active catalyst (Ni) from undergoing cycles of oxidation (forming inactive NiO) and reduction (forming Ni) as in the case of conventional regeneration technique.

## Conclusion

In conclusion, the reported work experimentally demonstrates the formation of MWCNTs in the novel CARGEN process. Additionally, a unique single step procedure to regenerate coked spent DRM/ CARGEN catalyst using CO_2_ as an activation media is also reported. A combination of the two processes (DRM/CARGEN + CO_2_ regeneration) provides a novel switching technique that utilizes CO_2_ during both operations as well regeneration cycles, proving to be a strong candidate for the commercialization of the DRM/CARGEN process that enables overall CO_2_ fixation.

## Supplementary Information

Supplementary Information.
