# Supplementary material for: A novel CO2 utilization technology for the synergistic co-production of multi-walled carbon nanotubes and syngas
Source: Sci Rep. 2021 Jan 14;11:1417. doi: 10.1038/s41598-021-80986-2 (PMC7809154; doi:10.1038/s41598-021-80986-2)
Supplement: Supplementary file 1 — Supplementary Information. [file 41598_2021_80986_MOESM1_ESM.docx]

A novel CO_2_ utilization technology for the synergistic co-production of multi-walled carbon nanotubes and syngas

Mohamed S. Challiwala^†,‡^, Hanif A. Choudhury^†^, Dingdi Wang^§^, Mahmoud M. El-Halwagi^‡^, Eric Weitz^§^_,_ Nimir O. Elbashir^*,†^

‡Artie Mcferrin Department of Chemical Engineering, Texas A&M University, College Station, USA

^€^TEES Gas & Fuels Research Center, College Station, USA

†Chemical Engineering and Petroleum Engineering Program, Texas A&M University at Qatar, Doha, Qatar

§Northwestern University, Evanston, Illinois, USA

*^(a)^Equal contribution from first two authors.*

Supporting information package

# CARGEN conceptualization and thermodynamic equilibrium assessment

This work provides first communication of a novel two-reactor in series processes; one targeted to capture CO_2_ and CH_4_ as solid carbon (we introduce this reactor as Carbon Generator Reactor or ‘CARGEN’), and the other to convert the remaining unconverted gases post CARGEN to syngas in a separate reactor (Combined reformer) in series. Figure S 1 in the main manuscript provides an illustration of this process and its potential product applications. The proposed scheme helps to convert significant quantities of CO_2_ to carbon at auto-thermal low temperature conditions (<500 ^o^C) in the first reactor of the two-reactor setup. The subsequent removal of solid carbon from the system (first reactor) enhances CO_2_ conversions to syngas in the second reactor tremendously by pushing the reaction forward thermodynamically, this removes the carbon from the system and is very beneficial from the CO_2_ life cycle assessment (LCA) perspective as demonstrated in our previous publication[1]. The initial phase of conceptualization of the CARGEN process was done via thermodynamic equilibrium estimation. The wisdom behind thermodynamic analysis was based on the knowledge that industrial reforming processes are operated at equilibrium conditions due to high temperature conditions and the need for high conversions[2]. At the same time, Gibb's free energy minimization is the easiest known method for quick reaction equilibrium calculations. More details on thermodynamic equations and calculations are provided in our previous publication [3]. Thermodynamic analysis of the results of operation of the second reactor shows that there is no carbon formation, which drives the reaction forward at much lesser energy requirements (approximately 50 kJ less) and at relatively lower temperatures compared to conventional reformer setups. A substantial increase in the syngas yield ratio is also obtained, which is not only beneficial for syngas production for Fischer Tropsch synthesis (requiring ~2:1 H_2_: CO ratio), but also for the hydrogen production (which requires high H_2_: CO ratios). In addition to the advantage of getting higher H_2_:CO ratio, significant increase in the methane and carbon dioxide conversion is also seen at much lower operating temperatures, which would otherwise be obtained only at higher temperatures (almost 250 ^o^C high) if conventional reforming setup is be used. The advantage of removing carbon in the first reformer helps in bringing down the operating temperature in the second reactor significantly, which is much more energy efficient than the conventional single reactor setup operated at higher temperatures to get similar levels of methane and carbon dioxide conversions at zero carbon deposition.

Figure S 1 below presents few of the case examples of the two-reactor CARGEN process demonstrating its flexibility of operation and effectiveness in enabling high CO_2_ conversions at low energy requirement conditions. Although both the cases have different feed composition and operational conditions, the energy requirements are approximately the same (about 120 kJ/mol) which is about 50% that of the dry reforming of methane (DRM) process. Further, the syngas ratio and the syngas yield of both the cases are different due to the differences in the feed composition of both the cases. Also, the overall CO_2_ conversion from the combined two reactor setup is around 65%. It should be noted that these results are not optimized yet and will be the subject of an independent future study.


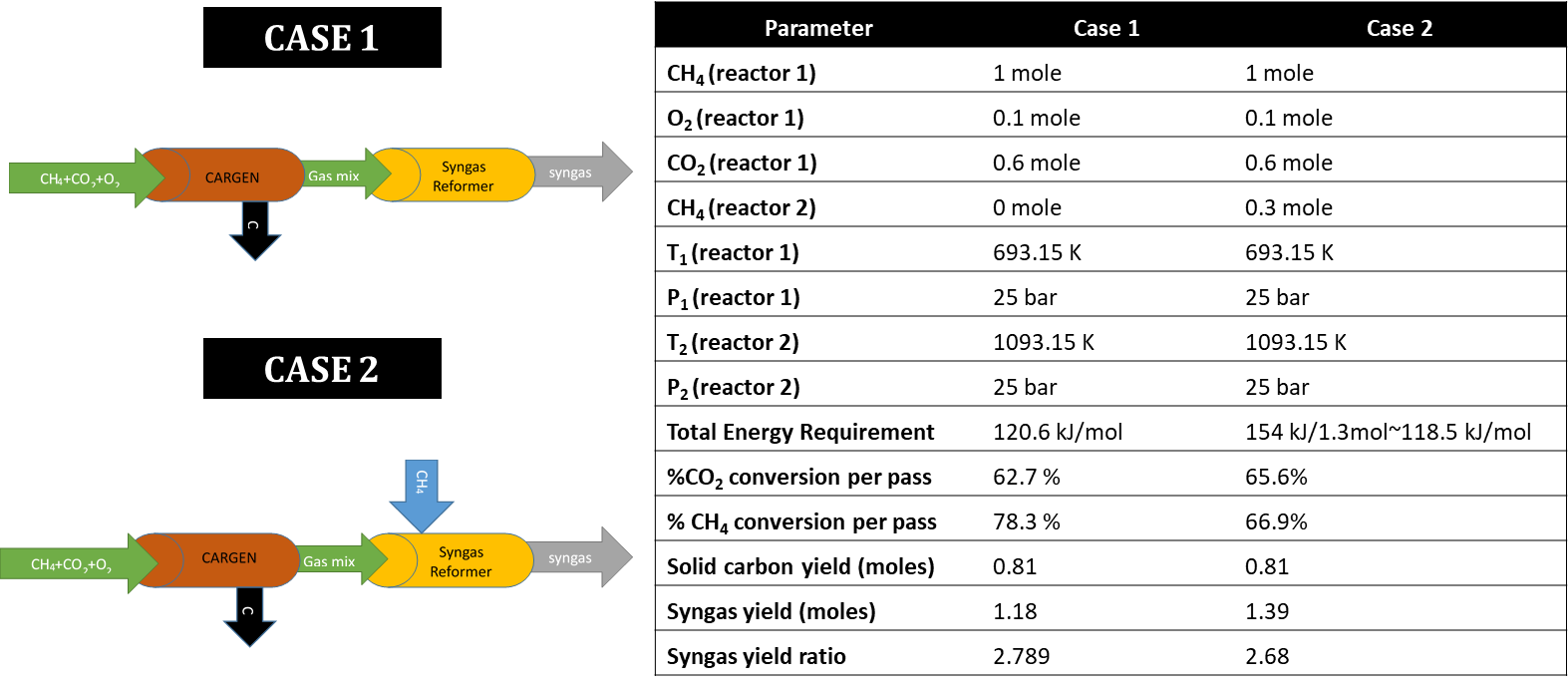


Figure S 1 Conceptual demonstration of the two reactor CARGEN process via thermodynamic equilibrium results

# Catalyst characterization

## Inductive coupled plasma (ICP) analysis

The Nickel catalyst used for all the studies in this paper was purchased from a commercial vendor with the labeled chemical composition of 20% Ni/γ-Al_2_O_3_. In order to deduce the exact composition, we have conducted an inductively coupled plasma (ICP) test using Plasma Quant PQ 9000 series ICP-OES device available in applied catalysis lab at Texas A&M University at Qatar (TAMUQ). Following is the tabular representation of the composition identified by this test:

Table S 1 ICP-OES data from Plasma Quant PQ 9000 instrument

| **sample name** | **expected Ni wt%** | **% Ni by ICP-OES** |
| --- | --- | --- |
| Riogen150-250 | 20 | 17.41667 |

## Temperature programmed reduction (TPR) analysis

In order to find the most suitable reduction conditions for the 20% Ni/γ-Al_2_O_3_ catalyst, a temperature programmed reduction (TPR) was conducted using a Autochem-II Micromeritics chemisorption equipment. The thermal conductivity detector (TCD) signal generation as a function of temperature is provided below in Figure S 2-a.


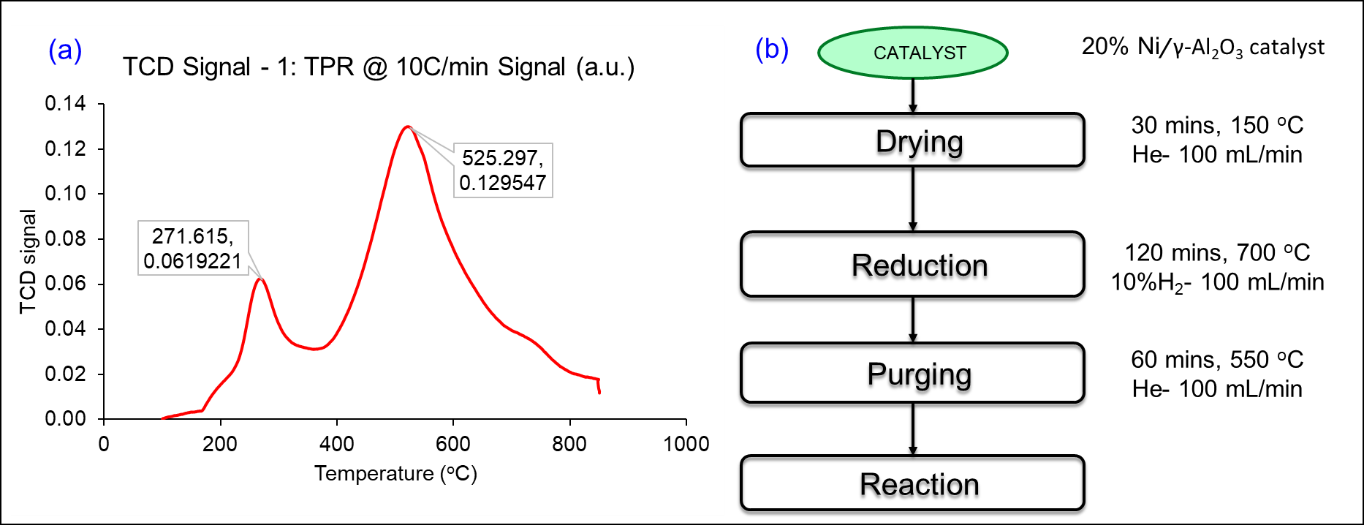


Figure S 2 (a) TPR profile of the commercial 20% Ni/γ-Al_2_O_3_ catalyst, (b) Experimental protocol used for all the reactions.

There are two observable peaks in the TPR profile at 271 °C and 525 °C temperature respectively and a further temperature increase does not result in further reduction. Therefore, a temperature beyond 600 C temperature will be sufficient for complete reduction. Nevertheless, in order to ensure complete catalyst activation, a temperature of 700 ^o^C was chosen for reduction. The reduction protocol for all the tests conducted in this study are provided in Figure S 2 b.

## Physisorption data

The physisorption data of the fresh catalyst sample was deduced using a standard Tri-star II Micromeritics instrument. Following table reports the key information from this study:

Figure S 3 BET and BJH plots of the commercial 20% Ni/γ-Al_2_O_3_ catalyst

Table S 2 Physisorption data of the fresh catalyst

| S.No | Quantity | Value |
| --- | --- | --- |
| 1 | Single point surface area at p/p^o^=0.30117 | 198.8170 m^2^/g |
| 2 | Brunauer-Emmett-Teller (BET) surface area | 201.44 m^2^/g |
| 3 | Barrett, Joyner, and Halenda (BJH) adsorption cumulative surface area of pores | 255.020 m^2^/g |
| 4 | BJH desorption cumulative surface area of pores | 325.5852 m^2^/g |
| 5 | BJH Adsorption cumulative volume of pores  between 5.000 Å and 1500.000 Å radius | 0.552825 cm^3^/g |
| 6 | BJH Desorption cumulative volume of pores  between 5.000 Å and 1500.000 Å radius | 0.585498 cm^3^/g |
| 7 | BJH Adsorption average pore radius (2V/A) | 43.355 Å |
| 8 | BJH Desorption average pore radius (2V/A) | 35.966 Å |

# Experimental analysis and quantification

Experiments conducted in this work were done using two reaction systems: (a) Thermo-gravimetric analysis (TGA) for weight gain measurement, and (b) Autochem II Micromeritics chemisorption equipment used as a flow through reactor. The quantification of results were done using material balance on the composition of the gases evolved during the reaction. The gaseous composition of various reaction species was detected using the standard residual gas analyzer (RGA) equipment (make HIDEN^®^ HPR20) calibrated before each run using calibration cylinder comprising of all the reaction gases (CH_4_, CO_2_, O_2_, H_2_, CO_2_, He and Ar). The following two subsections provide more details on both, the experimental procedure for each equipment as well as the quantification of results.

## Experimental procedure

### TGA analysis:

TGA analysis was conducted for weight gain testing and proof of concept studies of the CARGEN process. For this, the TGA/SDT Q600 equipment by TA^®^ at the applied catalysis laboratory of TAMUQ was used. All the experiments were done using approx. 20 mg of commercial 20% Ni/γ-Al_2_O_3_ catalyst at a constant gas composition of CH_4_/CO_2_/O_2_=1/0.6/0.1 for CARGEN and CH_4_/CO_2_=1:1 for DRM. Figure S 2 b describes standard experimental protocol followed for each test. Since the TGA equipment does not have a provision for extra mass flow controllers (MFCs), a specially designed bench with two calibrated mass flow controller(MFC) was used for mixing the two CARGEN cylinders (one flammable gas cylinder of methane and other of oxidizer gases CO_2_ and O_2_) as shown in Figure S 4. Both the MFCs were calibrated using a wet flow meter and calibration charts were developed for each reaction gas mixture for both the MFCs. In particular, both the MFCs were calibrated for 10% H_2_/90% Ar, 10% O_2_/90% He, 100% He, 100% Ar, 10% CH_4_/10% CO_2_/78% He/2% Ar, 100% N_2,_ 30% CO_2_/ 5% O_2_/ 63% He/2% Ar and 80% CH_4_/18%/He/2% Ar gases as they were required during different stages of the experiments. The mix gas of desired composition was fed to one of the two inlet ports of the TGA, while the second port was used for inert gas flow in between the reactions and for purging and drying operations. The TGA software enables design of methods based on desired protocol with the provision of switching of the gases at set time intervals while monitoring weight gain, while the exit of TGA is connected with HIDEN^®^ HPR 20 RGA for online monitoring of the reaction gas composition. The data obtained from TGA as well as RGA therefore allows simultaneous analysis of both, the weight gain as well as gaseous composition of the reaction gases. The raw data provided by RGA are in partial pressure values, which are then later processed in an in house developed Microsoft^®^ Excel^®^ VBA Macros^®^ code using a detailed material balance calculation to evaluate the overall CH_4_ and CO_2_ conversion.


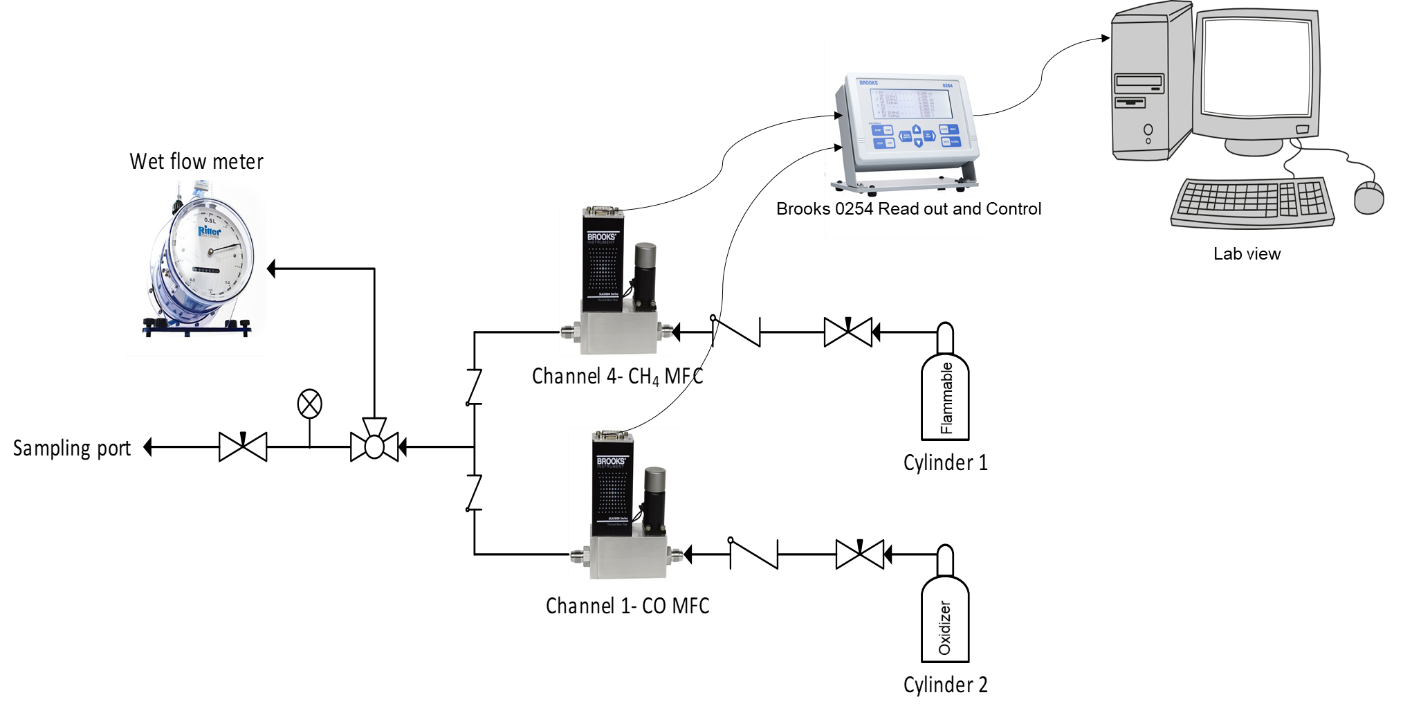


Figure S 4 Process flow diagram of a specially designed MFC bench for CARGEN and DRM testing

### Flow-through analysis

While the TGA instrument provides an estimate of the weight gain of carbon deposited during the CARGEN or DRM experiment, it does not allow sample analysis beyond 20-40 mg scale. The second limitation of TGA is its inability to provide 100% contact of reaction gases with the catalyst due to its “flow-top” nature as shown in Figure S 5-a. Therefore, in order to test the behavior of the system in an actual industrial type “flow-through” or packed bed kind of reaction system, the standard chemisorption equipment by Micromeritics® Autochem II equipment was used. This instrument has a provision to connect a “U” shaped reaction tube made up of quartz. The equipment also has several MFCs in its “carrier”, “prep” and “loop” modes that allow flow of the different reaction gases. In addition, the manual provided with the equipment provides an easy method for calculating the calibration factors of the various gas mixtures based on their thermal conductivity. Also, the equipment can be connected with standard RGA equipment, which in our case was HIDEN® RGA. The same bench of calibrated MFCs were used as in the case of TGA to minimize the number of parameter variations and also to follow the same protocol as described in Figure S 2-b. The main advantage of this instrument is that larger sample quantity can be analyzed. In our case, we have used about 200 mg of commercial 20% Ni/γ-Al2O3 catalyst for all testing using this instrument, which about 10 times more compared to the quantity used in the TGA, which also provides a quick testing of the behavior of the system under 10 times catalyst-scale up conditions.


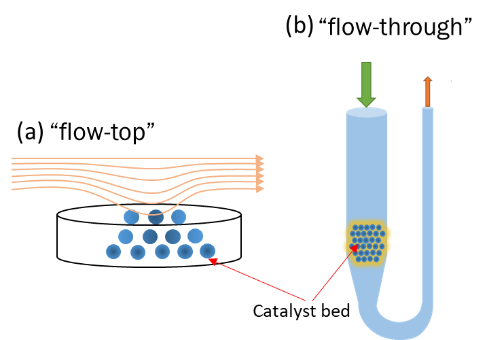


Figure S 5 Comparison of flow profile between (a) flow-top and (b) flow-through reaction systems

## Quantification

All the material balance conducted for both, the TGA as well as flow-through analysis was done using HIDEN® RGA as described in the previous sections. Material balance was done by comparing the quantities of reactant gases in the inlet and the outlet of reaction system using argon as an internal standard. A baseline reading of the inlet gas composition was taken before the experiment in bypass mode to identify the reactant gas composition. The exit gas composition was recorded at approx. every one minute interval in HIDEN® RGA. A material balance code was developed in Microsoft® Excel® VBA Macros® in order to quickly evaluate the conversion profiles. A case example of one data point calculation is provided below for further understanding.

**Material Balance calculations:**

Feed flow at STP condition shown by MFC:

Let C1 represent STP volume flow from cylinder containing CH_4_/He/Ar gas mix.

Let C2 represent STP volume flow from cylinder containing CO_2_/O_2_/He/Ar gas mix.

C1= 30 ml/min

C2= 48 ml/min

Total Flow of feed gas = V1= C1+C2= 78 ml/min.

*Table S 3 Feed gas composition shown by RGA (vol%) at* $120 ^{\circ}C$*:*

| H_2_ | 0 |
| --- | --- |
| He | 43.83 |
| CH_4_ | 30.11 |
| H_2_O | 0.35 |
| CO | 0 |
| CO_2_ | 22.32 |
| O_2_ | 2.24 |
| Argon | 1.67 |

As these readings were taken without reaction and there was no phase change in the system (water was not present), % volume distribution at STP and at RGA conditions $(at 120 ^{\circ}C)$ are same.

Volume of Argon ${(V}_{Ar,STP})$:

$V_{Ar,STP}=1.67 \% of 78\frac{mL}{min}$

$V_{Ar,STP}=1.3026\frac{mL}{min}$

$\rho_{Ar, STP}=1.7613 e^{-3}\frac{g}{mL}$ (Ref: ASPEN^®^ HYSYS V8.8, using PR fluid package)

Mass of Argon entering the system $(M_{Ar})$:

$M_{Ar}=V_{Ar,STP}\times\rho_{Ar,STP}$

$M_{Ar}=0.002294\frac{g}{min}$

As Mass of Argon will not change after the reaction, we will use Argon as internal standard.

Similarly,

Mass of methane, Carbon dioxide and Oxygen entering the system $(M_{CH_{4}}, M_{CO_{2}},M_{O_{2}})$ can be calculated as follows:

$M_{CH_{4},FEED}=V_{CH4,STP}\times\rho_{\mathrm{CH}4,STP}$

$\rho_{CH4,STP}= 7.0847 e^{-4}\frac{g}{mL}$ (Ref: ASPEN^®^ HYSYS V8.8, using PR fluid package)

$M_{CH_{4},FEED}=0.016639\frac{g}{min}$

$M_{CO2,FEED}=V_{CO_{2},STP}\times\rho_{\mathrm{CO}_{2},STP}$

$\rho_{CO_{2},STP}= 1.9518 e^{-3}\frac{g}{mL}$ (Ref: ASPEN^®^ HYSYS V8.8, using PR fluid package)

$M_{CO2,FEED}=0.03398\frac{g}{min}$

$M_{O2,FEED}=V_{O_{2},STP}\times\rho_{O_{2},STP}$

$\rho_{O_{2},STP}= 1.4109 e^{-3}\frac{g}{mL}$ (Ref: ASPEN^®^ HYSYS V8.8, using PR fluid package)

$M_{O2,FEED}=0.002465\frac{g}{min}$

*Table S 4 Product gas composition shown by RGA (vol%) at* $120 ^{\circ}C$*:*

|  | Product (Vol %) |
| --- | --- |
| H_2_ | 2.74 |
| He | 40.73 |
| CH_4_ | 26.09 |
| H_2_O | 6.39 |
| CO | 0.59 |
| CO_2_ | 21.7 |
| O_2_ | 0 |
| Ar | 1.7 |

Since Argon was taken as internal standard and as it does not take part in the reaction mixture, its mass will not change after reaction.

Therefore, volume of product gas mixture at RGA conditions $(120 ^{\circ}C)$ is as follows:

$V_{Ar,RGA}=\frac{M_{Ar}}{\rho_{Ar,RGA}}$

$\rho_{Ar,RGA}=1.2224 e^{-3}\frac{g}{mL}$ (Ref: ASPEN^®^ HYSYS V8.8, using PR fluid package)

$V_{Ar,RGA}=\frac{0.002294}{1.2224 e^{-3}}\frac{mL}{min}$

$V_{Ar,RGA}=1.8769\frac{mL}{min}$

Volume of Product gases at RGA condition:

$V_{products,RGA}=\frac{V_{Ar,RGA}}{Vol{\%}_{RGA}}$

$V_{products,RGA}=\frac{1.8769\times100}{1.7}\frac{mL}{min}$

$V_{products,RGA}=110.4051\frac{mL}{min}$

Therefore,

$V_{CH4,RGA}=Vol{\%}_{CH4,RGA}\times V_{products,RGA}$

$V_{CH4,RGA}=0.2609\times110.4051\frac{mL}{min}$

$V_{CH4,RGA}=28.8047\frac{mL}{min}$

Similarly, Volumes of $CO_{2},CO$ can be calculated as follows:

$V_{CO2,RGA}=0.217\times110.4051\frac{mL}{min}$

$V_{CO2,RGA}=23.9579\frac{mL}{min}$

$V_{CO,RGA}=0.0059\times110.4051\frac{mL}{min}$

$V_{CO,RGA}=0.6514\frac{mL}{min}$

Mass of $CH_{4}, CO_{2} \& O_{2}$ can be calculated by using densities of these gases at RGA conditions.

$M_{CH4,products}=\rho_{CH4,RGA}\times V_{CH4,RGA}$

$M_{CH4,products}=4.9117e^{-4}\times28.8047\frac{g}{min}$

$M_{CH4,products}=0.0141\frac{g}{min}$

Similarly,

$M_{CO2,products}=\rho_{CO2,RGA}\times V_{CO2,RGA}$

$M_{CO2,products}=1.3493 e^{-3}\times23.9579\frac{g}{min}$

$M_{CO2,products}=0.0323\frac{g}{min}$

$M_{CO,products}=8.5678 e^{-4}\times0.6514\frac{g}{min}$

$M_{CO,products}=0.0006\frac{g}{min}$

$M_{H2O}=5.5524 e^{-4}\times0.0639\times110.4051\frac{g}{min}$

$M_{H2O}=0.0039\frac{g}{min}$

$M_{H2}=6.1654 e^{-5}\times0.0274\times110.4051\frac{g}{min}$

$M_{H2}=0.0002\frac{g}{min}$

Therefore,

$CH_{4}\% conversion=\frac{M_{CH4,FEED}-M_{CH4,products}}{M_{CH4,FEED}}\times100$

$CH_{4}\% conversion=\frac{0.0166-0.0141}{0.0166}\times100$

$CH_{4}\% conversion=14.9709 \%$

Similarly,

$CO_{2}\% conversion=4.8646 \%$

**Atom balance for validation of results:**

Feed:

Moles of $CH_{4}$ entering the system $=\frac{M_{CH4,FEED}}{MW_{CH4}}=\frac{0.0166\frac{g}{min}}{16\frac{g}{gmol}}=0.0010\frac{gmol}{min}$

Moles of $CO_{2}$ entering the system $=\frac{M_{CO2,FEED}}{MW_{CO2}}=\frac{0.0340\frac{g}{min}}{44\frac{g}{gmol}}=0.0008\frac{gmol}{min}$

Moles of $O_{2}$ entering the system $=\frac{M_{O2,FEED}}{MW_{O2}}=\frac{0.0025\frac{g}{min}}{32\frac{g}{gmol}}=0.000077\frac{gmol}{min}$

Total $C moles$ entering the system=$Moles of C in CH_{4}+Moles of C in CO_{2}$

$=0.0010+0.00077$

$=0.00177 moles$

Total $O moles$ entering the system=$Moles of O in O2+Moles of O in CO_{2}$

$=2\times0.00077+2\times0.000077$

$=0.00167 moles$

Total $H moles$ entering the system=$Moles of H in CH_{4}$

$=4\times0.0010 moles$

$=0.004159 moles$

Product:

Moles of $CH_{4}$ leaving the system$=\frac{M_{CH4,products}}{MW_{CH4}}=\frac{0.0141\frac{g}{min}}{16\frac{g}{gmol}}=0.00088\frac{gmol}{min}$

Moles of $CO_{2}$ leaving the system$=\frac{M_{CO_{2},products}}{MW_{CO_{2}}}=\frac{0.0323\frac{g}{min}}{44\frac{g}{gmol}}=0.00073\frac{gmol}{min}$

Moles of $O_{2}$ leaving the system$=\frac{M_{O_{2},products}}{MW_{O_{2}}}=\frac{0\frac{g}{min}}{32\frac{g}{gmol}}=0\frac{gmol}{min}$

Moles of $H_{2}O$ leaving the system$=\frac{M_{H_{2}O,products}}{MW_{H_{2}O}}=\frac{0.0039\frac{g}{min}}{18\frac{g}{gmol}}=0.00022\frac{gmol}{min}$

Moles of $H_{2}$ leaving the system$=\frac{M_{H_{2},products}}{MW_{H_{2}}}=\frac{0.0002\frac{g}{min}}{2\frac{g}{gmol}}=0.000094\frac{gmol}{min}$

Moles of $CO$ leaving the system$=\frac{M_{CO,products}}{MW_{CO}}=\frac{0.0006\frac{g}{min}}{28\frac{g}{gmol}}=0.00002\frac{gmol}{min}$

Total $C moles$ leaving the system= $Moles of C in CH4+Moles of C in CO2+Moles of C in CO$

$=0.0009+0.0007+0.00002$

$=0.0018\frac{gmol}{min}$

Total $C moles$ accumulated in the reactor as solid carbon $= 0.0018-0.0016=0.0002\frac{gmoles}{min}$

Total $O moles$ leaving the system= $Moles of O in CO_{2}+Moles of O in CO+Moles of O in H_{2}O$

$=0.00073\times2+0.00002+0.00002+0.0002$

$=0.0017\frac{gmol}{min}$

Total $H moles$ leaving the system= $Moles of H in CH4+Moles of H in H2O+Moles of H in H_{2}$

$=0.00088\times4+0.00022\times2+0.000094\times2$

$=0.0046\frac{gmol}{min}$

**Validation of results:**

In order for Mass balance to be correct, total $O moles$ entering the system should be equal to total $O moles$leaving the system. Also, total $H moles$ entering the system should be equal to total $H moles$ leaving the system. $C moles$ will quantify carbon formation in the reactor as carbon is accumulated in the system.

Validation for $O moles:$

Total $O moles$ entering $=0.0017\frac{gmol}{min}$

Total $O moles$ leaving = $0.001727\frac{gmol}{min}$

Error $=\frac{0.0017-0.001727}{0.0017}\times100$ = -1.67 %

Validation for$H moles$:

Total $H moles$ entering $=0.00415975\frac{gmol}{min}$

Total $H moles$ leaving = $0.0041592\frac{gmol}{min}$

Error $=\frac{0.00415975-0.0041592}{0.00415975}\times100$ = 0.012688 %

Therefore, the overall error in mass balance calculated by the difference in the number of moles of hydrogen and oxygen entering and leaving the system is below 2% .

# X-ray Photoelectron Microscopy (XPS) and X-Ray Diffraction (XRD) analysis

To understand the difference between the conventional regeneration and the new regeneration technique, an experiment was conducted in which two similar batches of fresh catalysts were taken and subjected to DRM reaction with identical activation protocol. After a TOS of 4 hours, the first batch of the spent catalyst was subjected to the new regeneration procedure with 99% CO_2_ gas, while the second batch was subjected to a conventional regeneration procedure involving oxidation with 10% oxygen. The two catalysts were then subjected to the second cycle of operation, wherein the first batch in which O_2_ was used showed activity, while the second batch in which O_2_ was used did not. After this, both the spent samples were characterized by using ex-situ XRD and XPS analysis to identify their distinguishing features.

The XPS analysis was conducted using Axis Ultra DLD instrument by Kratos, a Shimadzu company located in the United Kingdom, in which a magnesium ($K\alpha)$ radiation of 1253.6 eV was operated at an emission current of 10 mA and 15 kV. First, a low resolution survey was conducted at a step size of 0.1 eV and a pass energy of 80 eV. Finally, all the peaks relative to C 1s, Ni 2p, O 1s and Al 2p were separately identified at high resolution with a pass energy of 5 eV and a step size of 1 eV. This analysis was conducted individually for spent DRM coked catalyst sample (Figure S 6-a), post CO_2_ TPO of DRM coked catalyst sample (Figure S 6-b), and post O_2_ TPO sample (Figure S 6-c). Table 3 provides surface elemental composition of C 1s, Ni 2p, O 1s and Al 2p of all the three samples.

Table S 5 Tabular representation of the XPS analysis surface composition of (a) spent coked DRM catalyst, (b) O_2_ TPO sample (c) CO_2_ TPO sample

| Name | 1. Spent coked DRM catalyst | | 1. O_2_ TPO sample | | 1. CO_2_ TPO sample | |
| --- | --- | --- | --- | --- | --- | --- |
|  | peak BE | atomic% | peak BE | atomic% | peak BE | atomic% |
| C 1s | 284.83 | 75.84 | 284.73 | 4.56 | 284.88 | 5.37 |
| Ni 2p | 855.15 | 0.62 | 855.27 | 3.02 | 855.94 | 2.01 |
| Al 2p | 74.42 | 8.21 | 73.29 | 31.41 | 73.47 | 30.81 |
| O 1s | 531.2 | 15.34 | 530.21 | 61.01 | 530.66 | 61.82 |

The comparison of surface carbon in samples a, b, and c, as shown in Table 3 above clearly demonstrates that both O_2_ TPO as well as CO_2_ TPO were able to reduce surface carbon from approx. 75% to approx. 5%. Further, the composition of other surface species; Ni, O, and Al, were almost same, revealing complete equivalency of both, the O_2_ TPO process, as well as CO_2_ TPO.

Since XPS is a surface analysis of the sample, XRD was done, which is a bulk analysis to further understand the extent of oxidation of the nickel catalyst using both the regeneration techniques. The XRD image analysis of this experiment is provided in Figure S7. As can be seen, XRD of the CO_2_ TPO treated catalyst is identical to that of a reduced catalyst indicating that the sample did not undergo any oxidation. While, the XRD pattern of O_2_ TPO treated sample showed the formation of NiO, which is inactive for the DRM reaction explaining why the catalyst wasn't active for the second cycle of operation. Further analysis of the XRD results using the Rietveld refinement method reveals that the O_2_ TPO sample had significant quantity of NiO (16.2 %) as opposed to 2.1% in CO_2_ TPO sample. Moreover, this quantity of NiO in O_2_ TPO sample is similar to that of reduced sample (1.3%).

Combined results of both, XRD as well as XPS conclusively establishes the soft oxidant characteristic of CO_2_ in selectively oxidizing the surface carbon while leaving the metallic state of pure nickel intact. On the other hand, since O_2_ is a strong oxidizing agent, it not only oxidizes the surface carbon but also the active metallic nickel sites to oxidized state which is inactive for reforming processes.


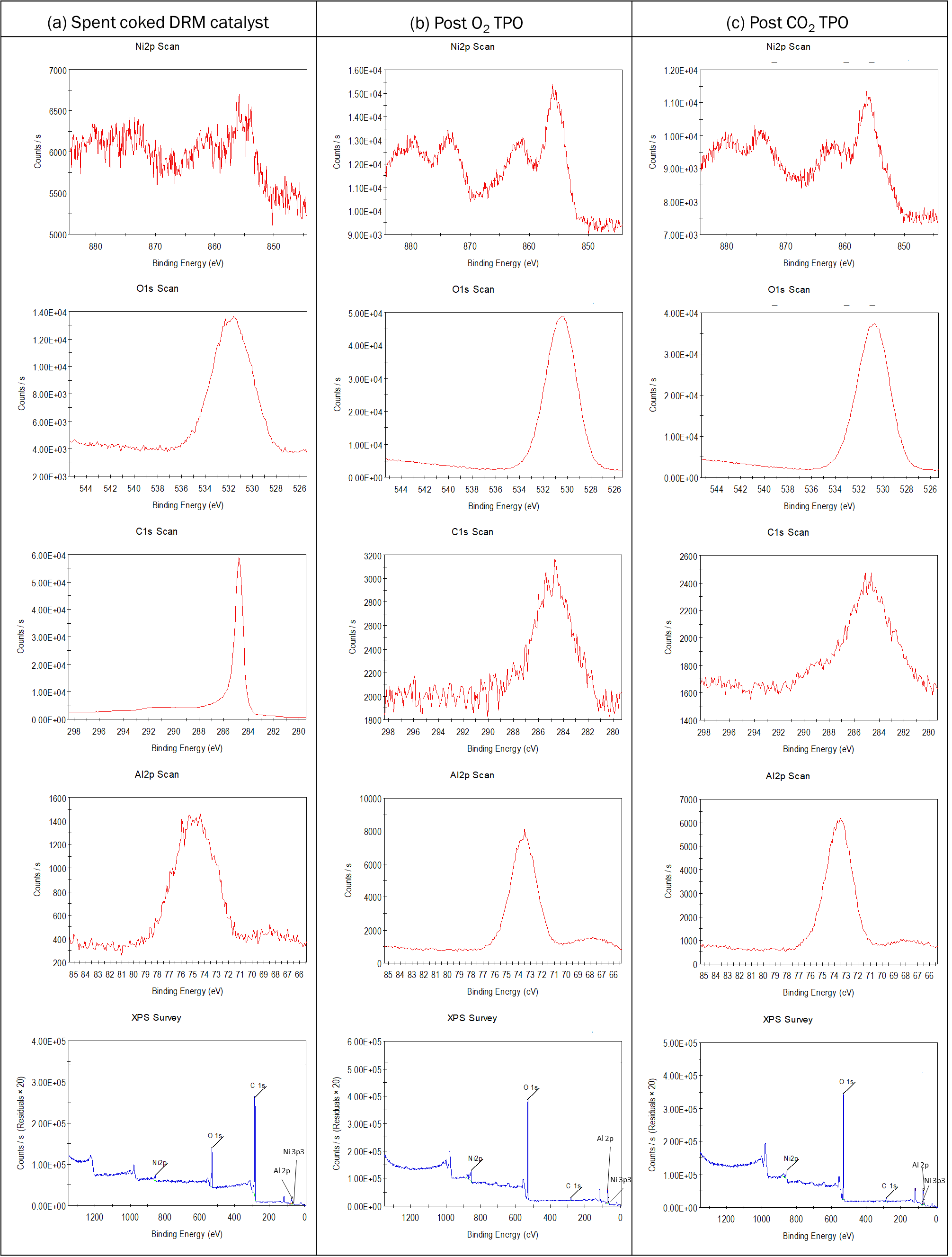


Figure S 6 XPS plots of (a) Spent coked DRM catalyst (b) Post O_2_ TPO sample (c) Post CO_2_ TPO sample


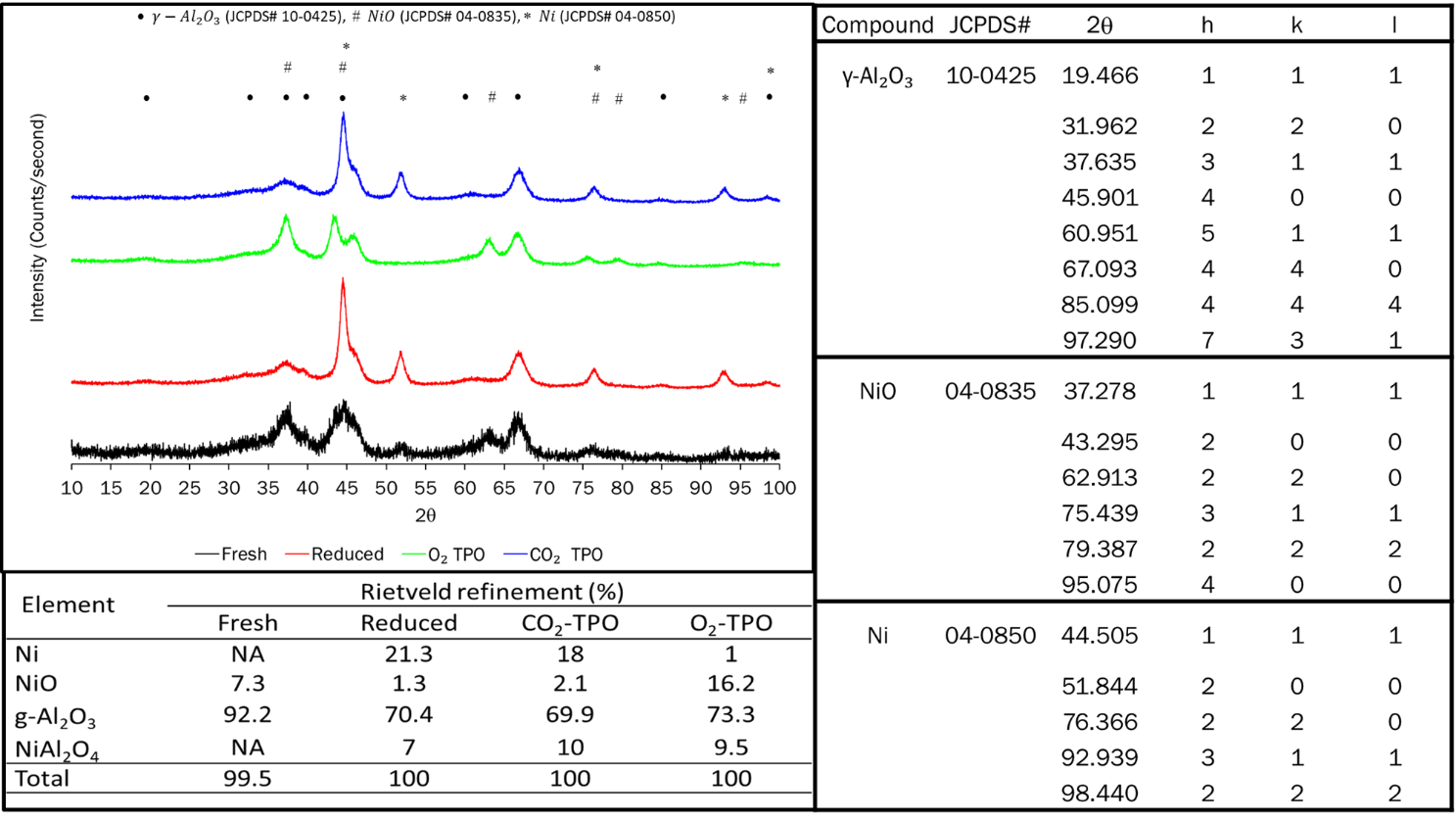


*Figure S 7 XRD plot of fresh 20% Ni/γ-Al_2_O_3_, reduced, post O_2_ TPO and post CO_2_ TPO samples with the JCPDS identification of the crystallographic phases and the Rietveld refinement composition comparison of each case.*

# CO_2_ footprint calculations

In order to compare the CO_2_ footprints of the new regeneration procedure and the conventional regeneration procedure, both direct and indirect CO_2_ emission and consumption information was accounted. For energy calculations, energy balance was conducted using the equilibrium moles calculated from gibbs free energy calculations. More details on the equilibrium assessment using Gibbs free energy calculation are provided in our previous publications[1], [3].It should be noted that 1 kg of surface carbon removal was considered as a basis for both the cases. Following step-by-step calculation provides details on this assessment:

Basis: 1 kg of surface carbon removal using CO_2_ TPO or O_2_ TPO process

Case 1:

Stoichiometric equation:

$CO_{2}+C\to2 CO \Delta H_{rxn, 700 ^{\circ}C}=118.13\frac{kJ}{mol}$ …. (Reaction 1)

$∵$ 1 kg carbon $\cong$ 83 moles …. (1)

$\therefore$ 83 moles of $CO_{2}$ will be utilized to convert 1 kg of carbon to 166 moles of $CO$ …. (2)

$∵$ $CO_{2}$ TPO is conducted at $700 ^{\circ}C$ temperature, by energy balance and using Gibbs free energy minimization, energy requirement at $700 ^{\circ}C$ for the stoichiometric reaction 1 = $9804.7\frac{kJ}{83 mol CO_{2}+83 mol C}$ …. (3)

If the energy requirement for step (3) is fulfilled by methane combustion (reaction 2 below) at 700$^{\circ}C$, the total number of moles of CO_2_ emitted by it will be 13.6 moles.

$CH_{4}+2O_{2}\to CO_{2}+{2H}_{2}O \Delta H_{rxn, 700 ^{\circ}C}=-721.36\frac{kJ}{mol}$ …. (Reaction 2)

Net $CO_{2}$ emission = $13.6-83=-69.4 moles\cong-3.05\frac{kg CO_{2} produced}{kg of surface carbon removed}$

Therefore, the total $CO_{2}$ fixation achieved= $3.05 \frac{kg CO_{2} produced}{kg of surface carbon removed}$

Case 2:

Stoichiometric equation:

$C+O_{2}\to CO_{2} \Delta H_{rxn, 700 ^{\circ}C}=-361.7489\frac{kJ}{mol}$ …. (Reaction 3)

$∵$ 1 kg carbon $\cong$ 83 moles …. (4)

$\therefore$ 83 moles of $CO_{2}$ will be produced per kg of surface carbon removed …. (5)

Carbon credits achieved from utilizing $-361.7489\frac{kj}{mol}\cong-30083\frac{kJ}{83 moles CO_{2} emitted}$= $\frac{30083}{721.3586}=41 moles$ …. (6)

Which means, if this energy was to be completely utilized while replace methane as the main source of heat, then the CO_2_ emission saved by burning 1 kg of carbon = $41$moles.

Total $CO_{2}$ emission= step (4)- step (6)

=$83-41=42\frac{moles}{kg of surface carbon removed}=0.49\frac{kg CO_{2} emitted}{kg surface carbon removed}$

Therefore, the total $CO_{2}$ emission= $0.49 \frac{kg CO_{2} produced}{kg of surface carbon removed}$

In summary, a simple comparison of the footprints associated with the CO_2_ TPO and O_2_ TPO, as demonstrated in cases 1 and 2 above, reveals that CO_2_ TPO is a net CO_2_ conversion process. At the same time, O_2_ TPO is a net CO_2_ emitting process. Also, the calculation above does not account for the footprint associated with the production of pure hydrogen, which is required for activating the post- O_2_ TPO catalyst as the active phase in the reforming process is metallic compound and not its oxidized state. It should be appreciated that the CO_2_ TPO does not oxidize the metallic sites, as it is not a potent oxidizing gas like O_2_, which along with carbon, also oxidizes the metallic catalyst. Further, the second benefit of this process is that the carbon monoxide produced from CO_2_ TPO could be used as a precursor for synthesizing a variety of chemicals and materials, which will bring additional economic benefit. Thirdly, the new procedure allows for reducing the downtime of the reactors, as it is a single step process compared to a dual step conventional regeneration procedure.

# Citations

[1] M. Challiwala, S. Afzal, H. A. Choudhury, D. Sengupta, M. El-halwagi, and N. O. Elbashir, “Alternative pathways for CO2 utilization for enhanced methane dry reforming technology,” in *Advances in Carbon Management Technologies*, 2019.

[2] M. M. B. Noureldin, N. O. Elbashir, and M. M. El-Halwagi, “Optimization and Selection of Reforming Approaches for Syngas Generation from Natural/Shale Gas,” *Ind. Eng. Chem. Res.*, vol. 53, no. 5, pp. 1841–1855, 2014.

[3] M. S. Challiwala, M. M. Ghouri, P. Linke, M. M. El-Halwagi, and N. O. Elbashir, “A combined thermo-kinetic analysis of various methane reforming technologies: Comparison with dry reforming,” *J. CO2 Util.*, vol. 17, pp. 99–111, 2017.
